# Baseline Age Disparities Across GLP‐1 Receptor Agonist–Based Trials

**DOI:** 10.1111/1753-0407.70283

**Published:** 2026-10-18

**Authors:** Wenjia Yang, Wei Zhao, Xiaoling Cai, Linong Ji

**Affiliations:** ^1^ Department of Endocrinology and Metabolism Peking University People's Hospital Beijing China

To the Editor,

1

Glucagon‐like peptide‐1 receptor agonist (GLP‐1 RA)–based therapies now span indications from glycemic control to weight management, and cardiovascular and renal risk reduction [1]. As trials have progressed along this translational arc, the enrolled populations may differ substantially. We examined whether baseline age varies systematically by primary endpoint category across the published GLP‐1 RA–based randomized controlled trial (RCT) literature.

We included published RCTs of 11 GLP‐1 RA–based agents across five pharmacologic classes: single‐target GLP‐1 RAs (liraglutide, semaglutide, exenatide, lixisenatide, albiglutide, and dulaglutide), a glucose‐dependent insulinotropic polypeptide (GIP)/GLP‐1 dual agonist (tirzepatide), glucagon receptor (GCGR)/GLP‐1 dual or triple agonists (mazdutide and retatrutide), an amylin/GLP‐1 co‐agonist (cagrilintide–semaglutide), and an oral small‐molecule GLP‐1 RA (orforglipron). Trials were categorized by primary endpoint: glycemic efficacy, weight‐loss efficacy, or clinical outcomes (major adverse cardiovascular events [MACE] or renal composite). Baseline age (mean ± standard deviation [SD]) was extracted from each publication. Pooled estimates were obtained via a DerSimonian–Laird random‐effects model.

A total of 144 RCTs encompassing 189 466 participants were included. Pooled baseline age was 56.4 years (95% confidence interval [CI], 55.7 to 57.1) for glycemic‐efficacy trials, 46.7 years (95% CI, 44.2 to 49.3) for weight‐loss trials, and 64.8 years (95% CI, 63.4 to 66.2) for outcome trials. Compared with weight‐loss trials, pooled baseline age was 9.7 years (95% CI, 7.0 to 12.3) higher in glycemic‐efficacy trials and 18.1 years (95% CI, 15.2 to 21.0) higher in outcome trials. Pooled baseline age was also 8.4 years (95% CI, 6.9 to 10.0) higher in outcome trials than in glycemic‐efficacy trials. Pairwise differences were all significant (*p* < 0.001 for each comparison) (Figure 1).

**FIGURE 1:**
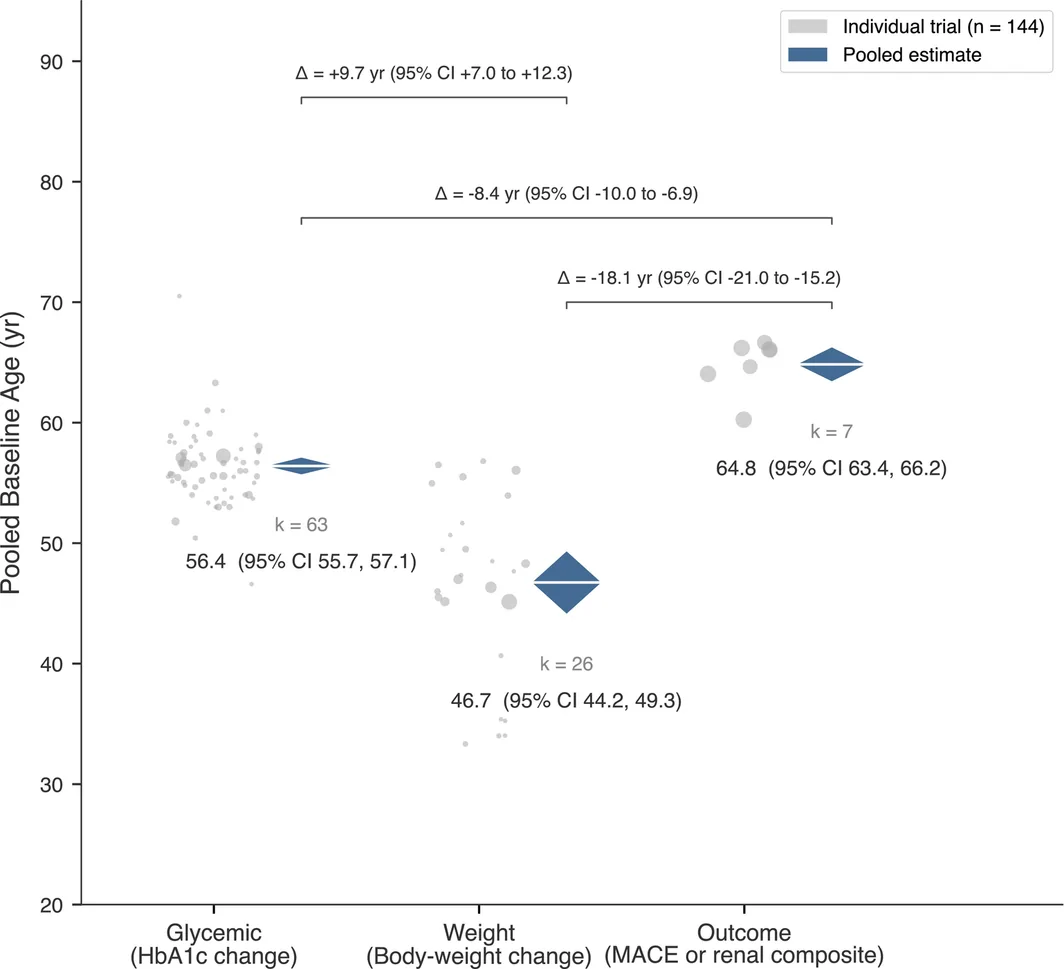
Pooled baseline age across GLP‐1 receptor agonist–based randomized controlled trials, grouped by primary endpoint category.

These findings reveal a structural gap in the evidence supporting GLP‐1 RA–based therapies: three largely non‐overlapping age strata are being studied, yet clinical decisions apply across the entire age spectrum. This approximately 18‐year gradient carries implications for the generalizability of trial findings. The efficacy and safety observed in weight‐loss trials may not translate to older adults [2, 3]. Conversely, the cardiorenal protection demonstrated in outcome trials—where the mean age exceeds 64 years—does not directly inform the risk–benefit calculus in younger patients initiating these agents for weight loss or glycemic control.

The differences in pooled baseline age across endpoint categories highlight a broader opportunity for the design of metabolic disease trials. Ensuring that enrollment spans a wider age range—or that age‐stratified analyses are prespecified—would allow the evidence to speak to the patients who actually receive these treatments in practice.

## Author Contributions

All authors have contributed significantly to this work and meet the authorship criteria in accordance with the latest guidelines of the International Committee of Medical Journal Editors (ICMJE). Wenjia Yang contributed to conceptualization, methodology, formal analysis, investigation, data curation, and writing – original draft. Wei Zhao contributed to methodology, validation, formal analysis, investigation, and writing. Xiaoling Cai contributed to conceptualization, methodology, supervision, and review. Linong Ji contributed to conceptualization, supervision, funding acquisition, project administration, and writing – review and editing. All authors have read and approved the final version of the manuscript and agree to be accountable for all aspects of the work.

## Funding

This work is funded by the 2024 National Clinical Key Specialty Construction Program of China (Department of Endocrinology, Peking University People's Hospital) with support from the central government budget.

## Conflicts of Interest

The authors declare no conflicts of interest.

## Data Availability

The data that support the findings of this study are available on request from the corresponding author. The data are not publicly available due to privacy or ethical restrictions.
